# Development of real‐time motion verification system using in‐room optical images for respiratory‐gated radiotherapy

**DOI:** 10.1120/jacmp.v14i5.4245

**Published:** 2013-09-06

**Authors:** Yang‐Kyun Park, Tae‐geun Son, Hwiyoung Kim, Jaegi Lee, Wonmo Sung, Il Han Kim, Kunwoo Lee, Young‐bong Bang, Sung‐Joon Ye

**Affiliations:** ^1^ Department of Radiation Oncology Seoul National University Hospital Seoul; ^2^ Interdisciplinary Program in Radiation Applied Life Science Seoul National University Seoul; ^3^ Department of Mechanical and Aerospace Engineering Seoul National University Seoul; ^4^ Program in Biomedical Radiation Sciences Department of Transdisciplinary Studies Graduate School of Convergence Science and Technology Seoul National University Seoul; ^5^ Advanced Institutes of Convergence Technology Seoul National University Suwon

**Keywords:** gated radiotherapy, external marker tracking, quality assurance, 4D CT

## Abstract

Phase‐based respiratory‐gated radiotherapy relies on the reproducibility of patient breathing during the treatment. To monitor the positional reproducibility of patient breathing against a 4D CT simulation, we developed a real‐time motion verification system (RMVS) using an optical tracking technology. The system in the treatment room was integrated with a real‐time position management system. To test the system, an anthropomorphic phantom that was mounted on a motion platform moved on a programmed breathing pattern and then underwent a 4D CT simulation with RPM. The phase‐resolved anterior surface lines were extracted from the 4D CT data to constitute 4D reference lines. In the treatment room, three infrared reflective markers were attached on the superior, middle, and inferior parts of the phantom along with the body midline and then RMVS could track those markers using an optical camera system. The real‐time phase information extracted from RPM was delivered to RMVS via in‐house network software. Thus, the real‐time anterior‐posterior positions of the markers were simultaneously compared with the 4D reference lines. The technical feasibility of RMVS was evaluated by repeating the above procedure under several scenarios such as ideal case (with identical motion parameters between simulation and treatment), cycle change, baseline shift, displacement change, and breathing type changes (abdominal or chest breathing). The system capability for operating under irregular breathing was also investigated using real patient data. The evaluation results showed that RMVS has a competence to detect phase‐matching errors between patient's motion during the treatment and 4D CT simulation. Thus, we concluded that RMVS could be used as an online quality assurance tool for phase‐based gating treatments.

PACS number: 87.55.Qr

## I. INTRODUCTION

Respiration‐induced motion has been a significant challenge in radiotherapy for thoracic and abdominal tumors.^(1)^ To manage this motion, the respiratory gating technique was introduced and evaluated in previous studies.^(^ ^2^ ^,^ ^3^ ^)^ In this technique, radiation is controlled by a beam delivery system within a particular portion of the patient's breathing cycle (so‐called gating window).^(4)^ The tumor motion only within the gating window is taken into account in both treatment planning and delivery processes. Therefore, with this technique tumor margins can be reduced and, thus, tumor dose escalation is enabled without compromising normal tissue sparing.^(5)^

One widely used gating system with external marker‐based monitoring is the real‐time position management (RPM) system (Varian Medical Systems, Palo Alto, CA). Several studies have been performed to evaluate efficacy of RPM.^(^ ^6^ ^,^ ^7^ ^)^ The RPM system provides two alternative methods to define the gating window: phase‐based gating and amplitude‐based gating.^(8)^ It has been reported that amplitude‐based gating results in lower residual motion than phase‐based gating.^(^ ^7^ ^,^ ^9^ ^)^ However, in some institutions, phase‐based gating is preferred for two reasons: (1) phase‐based gating provides a stable duty cycle, whereas the amplitude‐based gating suffers from baseline shifts;^(^ ^3^ ^,^ ^9^ ^)^ and (2) some specific CT systems correlate images only in terms of the respiration phase.^(^ ^8^ ^,^ ^10^ ^)^

In the RPM phase‐based gating technique, the reproducibility of respiratory motion (e.g., displacement according to the respiratory phase) between simulation and treatment fractions is essential. However, in routine clinical practice, the RPM system with a phase‐based mode has not provided any solution to quantitatively compare two displacements during the delivery and the CT simulation. The best way to verify the reproducibility of the respiratory motion is to use X‐ray imaging,^(4)^ which results in excessive radiation exposure if the acquisitions are performed frequently during treatment.

To verify the reproducibility of the external marker position, several methods using noninvasive optical tracking were proposed. Wong et al.^(11)^ used the ExacTrac system (BrainLAB AG, Feldkirchen, Germany) to monitor a patient's abdominal surface positions during the deep inhalation breath‐hold (DIBH) technique. Venkat et al.^(12)^ developed an audiovisual biofeedback system using a single infrared (IR) reflective marker to improve and verify the reproducibility of external marker positions between simulation and treatment. On the other hand, Plathow et al.^(13)^ demonstrated that the correlation between internal tumor motion and external marker motion was highly dependent on the breathing type such as abdominal breathing and thoracic breathing. This finding supported the idea that motion monitoring with a single external marker could not provide sufficient tracking information for tumor motion.^(^ ^14^ ^,^ ^15^ ^)^ Therefore, to improve the internal‐external correlation, several studies have proposed multiple external marker tracking^(16)^ or markerless surface monitoring^(17–19^ rather than single external marker tracking. For tracking of multiple markers or a patient's surface, commercial products such as ExacTrac and GateRT (VisionRT, London, UK) are available on the market. However, so far no study has attempted to use 4D CT data as the reference of motion monitoring to check the positional reproducibility of the multiple external markers or patient's external surface.

This study aimed to develop a quality assurance technique to quantitatively compare a patient's external surface motion between 4D CT simulation and treatment for RPM phase‐based gating. The developed technique involved stereocamera‐based optical tracking, surface extraction from 4D CT simulation data, and a phase synchronization method with RPM. Phantom experiments were performed using a programmable respiratory motion platform to evaluate the performance of our system.

## II. MATERIALS AND METHODS

### A. System overview

**Figure 1:**
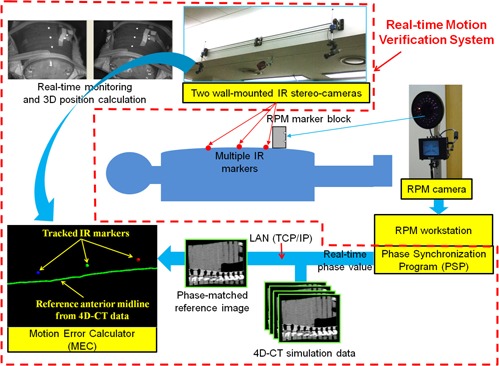
A schematic representation for the developed quality assurance method using real‐time motion verification system (RMVS).

A schematic illustration of the proposed quality assurance method for RPM phase‐based gating is shown in Fig. 1. A conventional RPM‐based gating technique uses a single IR camera, an IR reflective marker bock, and a workstation connected to the beam delivery system. In order to acquire real‐time images of patient surface motion according to the signals of the RPM system, the developed system consisted of two wall‐mounted stereocameras and multiple IR markers on the patient's anterior surface, and a phase synchronization program (PSP). The motion error calculator (MEC) was programmed to quantitatively compare acquired real‐time images of the multiple IR markers with phase‐matched reference images extracted from 4D CT simulation data. Details of the developed system are given in the Materials & Methods Sections C, D, E and F below.

### B. Anthropomorphic phantom and motion platforms

An anthropomorphic phantom (Alderson Research Laboratories, New York, NY) and two different types of motion platform (A and B) were used to evaluate the system. Platform A was composed of an acrylic stage and two linear actuators that were approximately 40 cm apart along the superior‐inferior direction. The actuators oscillated the stage between two positions in the anterior‐posterior direction. The two oscillation positions and the cycle can be programmed. In normal mode, these two actuators were synchronized to keep the stage horizontal. However, by fixing one actuator in a certain position, the motion platform can also simulate asymmetric motions, such as abdominal and chest breathing. On the other hand, platform B is a fully programmable motion platform which can simulate arbitrary motions in 3D space, such as patient respiratory data. The platform is composed of polycarbonate panels and four stepping motors to simulate 3D tumor and external marker motion. The positional accuracy of the platform had been evaluated by using a high‐resolution laser sensor (RF603, RIFTEK, Minsk, Belarus), of which spatial resolution is 0.03 mm and temporal resolution is 0.01 ms. It was determined to be 0.2 mm.^(20)^ In this study, most of the phantom experiments were performed with platform A because of its simplicity in operation and unique feature of asymmetric motions for breathing type change simulations. Platform B was used to evaluate the accuracy of the optical tracking system and to simulate real patient respiratory data.

### C. Stereocamera system and IR marker tracking

An IR‐based stereocamera enabled us to monitor the phantom's respiratory motion in real time. The system hardware consisted of two charge‐coupled device (CCD) cameras (HVR2300C, Hi Vision System, Korea) with universal serial bus (USB) 2.0 interface, 2 IR filters (B+W 092, Schneider‐Kreuznach, Bad Kreuznach, Germany), multiple IR light‐emitting diodes (LEDs), and a personal computer (PC) with a 2.8 GHz central processing unit (CPU). A custom‐fabricated frame housing the cameras, IR filters, and IR LEDs was mounted on the inferior wall of the treatment room. The stereocamera system was calibrated with a checkerboard template and free software (Camera Calibration Toolbox, Imperial College, London, UK). The calibration procedures were performed by following Zhang's method.^(21)^ Three IR reflective markers (Scotchlite 154 TM 3000X, 3M, St. Paul, MN) having a diameter of 6 mm were attached on the phantom surface along the body midline, even though the system can track multiple external markers simultaneously independent of their positions and number. In our previous studies using the same marker tracking method, the tracking accuracy was found to be 0.4 ± 0.4 mm and 0.2 ± 0.4 mm for 3D and vertical direction, respectively.^(^ ^22^ ^,^ ^23^ ^)^

### D. Extraction of 4D CT‐based reference lines

**Figure 2:**
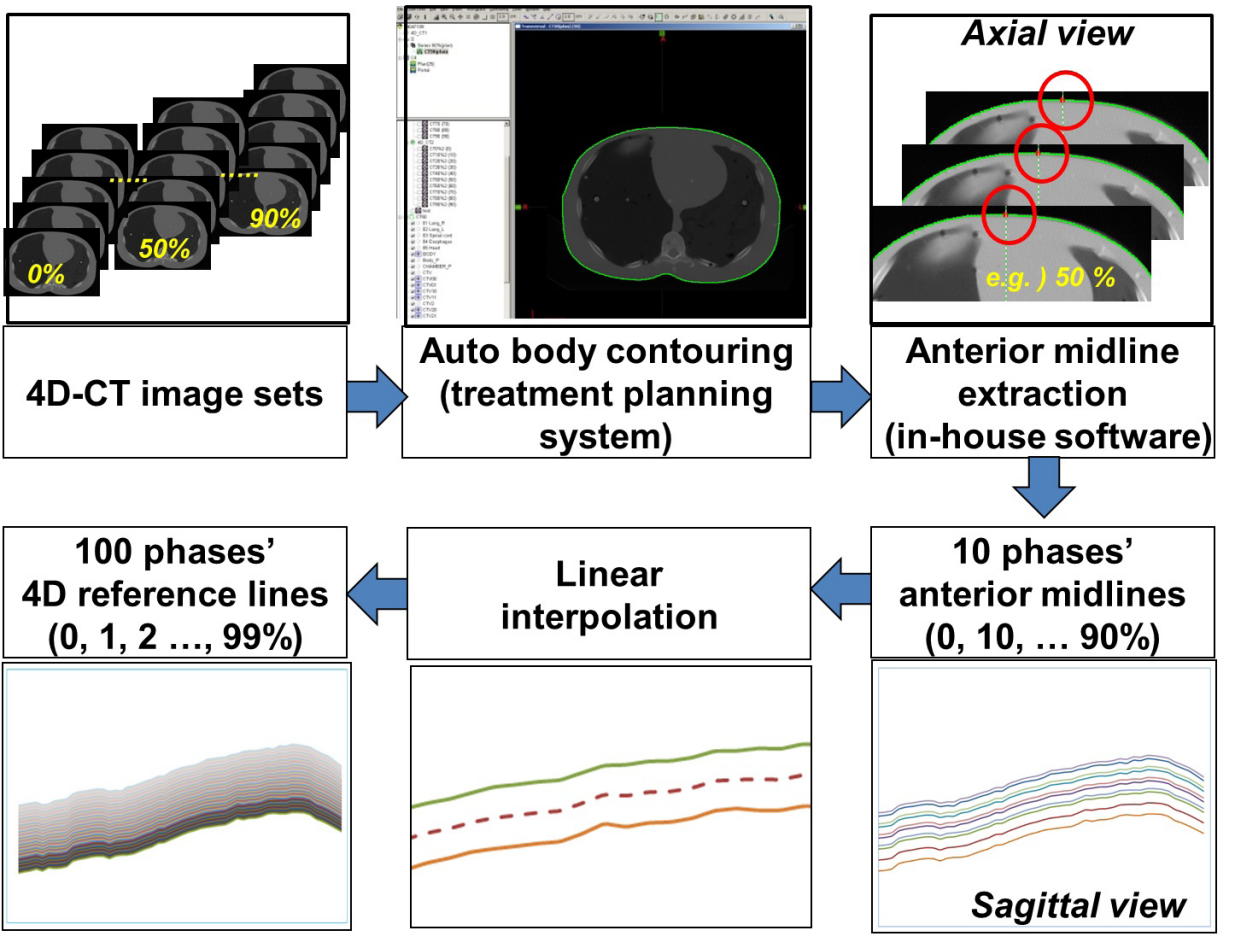
A flow chart demonstrating how to generate the 4D reference lines from 4D CT simulation data.

Phase‐resolved anterior body midlines were extracted from the 4D CT simulation data and used as vertical displacement references denoted as “4D reference lines”. A workflow to obtain the 4D reference lines is shown in Fig. 2. Ten phase image sets of the phantom were acquired from a 4D CT scanner (Big Bore Brilliance, Philips Medical Systems, Bothell, WA) equipped with the RPM system. The acquired images were transferred to a treatment planning system (Eclipse, Varian Medical Systems, Palo Alto, CA), and external body surfaces were automatically contoured in the system by using a CT number threshold of ‐450 HU. These body contours were then exported into DICOM‐RT structure (RS) files. Using in‐house DICOM processing software, ten sets of anterior surface midlines tagged by unique respiratory phase values were extracted from the RS files. The initially generated ten sets of body midlines were linearly interpolated to create 100 sets of data so that each line was assigned integer phase values ranging from 0 to 99. These 100 body midlines were defined as “4D reference lines” in this study. Finally, the complete sets of the 4D reference lines were exported to a text file.

### E. Respiratory phase synchronization with RPM

**Figure 3:**
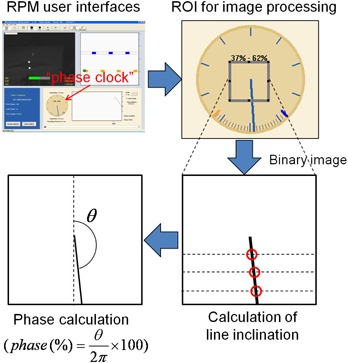
An illustration of the procedure to acquire the respiratory phase value from the RPM workstation. The PSP processed the ROI set on the “phase clock” and transferred the calculated phase value to the RMVS over a network in real time.

In RPM‐phase based gating, beam‐on and ‐off are controlled by the RPM real‐time calculated phase information. It has been reported that the real‐time calculated phase is error‐prone and the retrospective phase calculation using a RPM log file (called “vxp file”) is more accurate.^(8)^ However, despite of its imperfectness, the real‐time calculated phase was our choice for the online verification for detecting any errors by monitoring in real time the relationship between phase and displacement. Therefore, to compare current positions of the tracked markers with the reference line at the same phase taken from the 100 sets of 4D reference lines, the phase synchronization program (PSP) was developed to provide this phase information in real time. The PSP was installed on the RPM workstation and operated simultaneously with RPM software version 1.7.5. As the RPM software provided a clock‐shaped interface for displaying respiratory phase values, the PSP set the ROI at the center of the “clock” and processed the image of the ROI in real time, as shown in Fig. 3. Finally, the phase value calculated by the software was then transferred to the RMVS through a LAN.

### F. Motion error calculator

The motion error calculator (MEC) is a software module integrated into the RMVS. Three types of input data were required to run the MEC. The first data were the 3D positions of the external markers tracked by the stereocamera system in real time, the second was the 4D reference lines, and the third was the current phase value acquired from the PSP in real time. First, the MEC imports a text file containing the 100 sets of 4D reference lines. Secondly, current 3D position data of the tracked external markers were acquired by the stereocamera system in real time and simultaneously transferred into the MEC. Thirdly, the MEC received the RPM‐generated current phase information from PSP and dynamically selected the reference line corresponding to the current phase value. Finally, displacement error between the external marker positions and the reference line was calculated and displayed on an operator's monitor screen in real time. Even though the stereocamera system can track 3D coordinates of the markers, only the z‐axis value was used to calculate errors. The x‐axis value was not used because the 4D reference line was assumed to be a body midline having the x‐axis value of 0. On the other hand, the y‐axis value was used to find the corresponding projected points of the tracked marker on the 4D reference line. The MEC calculated singed and absolute errors, which are given as follows:

(1) $$Signed\;error(\Delta_{i})=P_{i}{\left(t\right)}_{z}-z_{4DCT}(\phi (t),P_{i}{\left(t\right)}_{y}$$

(2) $$Absolut\text{ee}rror=\left|\Delta_{i}\right|$$

where *i* is the marker index, Δ is the signed error for $i^{\text{th}}$ marker, ${P_{i}(t)}_{y}$ and ${P_{i}(t)}_{2}$ are the y and z component of the 3D position of $i^{\text{th}}$ marker at time t, respectively. $z_{4\text{DCT}}$ is the anterior‐posterior position of the 4D reference lines, and φ(t) is the integer value of the respiratory phase at time *t*. The average error for all markers can be defined as an overall error as follows:

(3) $$Overal\text{le}rror(signed)=\frac{1}{N}\sum_{i=1}^{N}\Delta_{i}$$

(4) $$Overal\text{le}rror(absolute)=\frac{1}{N}\sum_{i=1}^{N}\left|\Delta_{i}\right|$$

where *N* is the number of markers. If the motion error of individual markers exceeds a user‐defined tolerance, the MEC can display a warning message to the operator.

The proposed point‐to‐line matching method allowed us to remove the necessity of an external marker during 4D CT scan. Even though a corresponding projected point on the reference line is not exactly matched with a tracked point (especially when considering deformation of patient surface), the proposed error metrics can provide quantitative error values when there are some problems in positional reproducibility between simulation and treatment.

### G. Phantom evaluation

#### G.1 Evaluation of system accuracy and precision

Prior to the phantom experiments with various motion parameters, the overall accuracy and precision of the system was evaluated. Even though the accuracy of the stereocamera system and platform B had been evaluated in our previous studies, a test experiment with an irregular breathing pattern was performed by using both stereocamera and platform B. Three IR markers on the RANDO phantom (The Phantom Laboratory, Salem, NY) were tracked in this experiment. On the other hand, to evaluate the accuracy of platform A, a test motion with a displacement of 31 mm and a cycle of 3.1 s was simulated with four equidistant markers and solid water slabs (one more marker and flat surface phantom in this initial evaluation). The motion parameters were then compared with the tracking data acquired by the stereocamera system. Using the test motion, the accuracy of RPM and 4D CT was also evaluated for the comparison purpose.

Finally, the accuracy of phase synchronization was evaluated by comparing the respiratory phase data of RPM and RMVS to confirm that both systems have the same phase value when the same positional data is given.

#### G.2 Evaluation of interfractional changes in breathing motion

**Figure 4:**
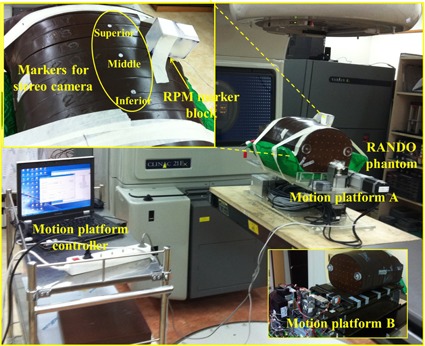
Experimental setup for system evaluation on a motion phantom. Two types of motion platform were used for the simulations of breathing type changes (platform A) and irregular breathing (platform B).

**Figure 5:**
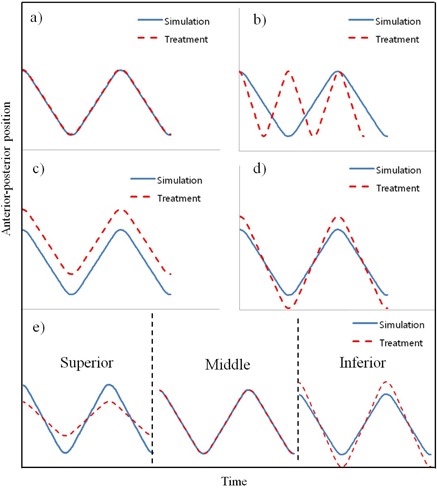
Five motion scenarios were taken into account in the phantom experiments: (a) ideal, (b) cycle change, (c) baseline shift, (d) displacement change, and (e) breathing type change.

Three IR reflective markers in an interval of 6 cm were attached on the RANDO phantom surface (superior, middle, and inferior markers), and a RPM marker block was placed beside the middle marker. The phantom (mounted on platform A) oscillated regularly according to a reference motion pattern (“normal breathing” hereafter, for convenience) having a displacement of 20 mm, and a cycle of 3.1 s for all three markers. The phantom then underwent a 4D CT scan and 4D reference lines were prepared as described in Section D above. In a treatment room, various motion scenarios were simulated to test whether our system could detect the abnormalities when the motion pattern was changed from that of CT simulation. Figure 4 shows the experimental setup in the treatment room. Five different motion scenarios were designed to simulate possible clinical situations. They included ideal (identical motion parameters between CT simulation and treatment), cycle change, baseline shift, displacement change, and breathing type change cases. Figure 5 shows the five motion scenarios graphically. In the ideal case, motion errors were expected to be zero. Even in the cycle change scenario, no motion errors were expected because the RPM phase‐based system cannot account for any systematic changes in the breathing cycle. In contrast, the baseline shift, displacement change, and breathing type change scenarios were expected to exhibit significant motion errors in our developed system. In total, eight sets of phantom experiments, the parameters of which are listed in Table 1, were performed in the treatment room. Each experiment took 100 s. Mean signed errors (MSEs) and mean absolute errors (MAEs) between external marker positions and 4D reference lines were evaluated for each experiment.

**Table 1:** Various motion parameters of the phantom experiments

				*Displacement (mm)*			
*Scenario*	*Experiment No*.	*Description*	*Baseline (mm)*	*Superior*	*Middle*	*Inferior*	*Cycle (s)*
Ideal case	1	Ideal case	0	20	20	20	3.1
Cycle change	2	Longer cycle	0	20	20	20	4.7
Baseline shift	3	Offset ‐5	‐5	20	20	20	3.1
	4	Offset +5	5	20	20	20	3.1
Displacement change	5	Smaller displacement	0	10	10	10	3.1
	6	Larger displacement	0	40	40	40	3.1
Breathing type change	7	Abdominal breathing	0	14.6	20	25.4	3.1
	8	Chest breathing	0	25.4	20	14.6	3.1

#### G.3 Evaluation of intrafractional changes in breathing motion

Evaluation for intrafractional motion changes was simply performed by using a combination of phantom motion sequences including normal breathing, abdominal breathing, chest breathing, offset ‐5 mm, and larger displacement patterns. Approximately 60 s was allotted for each sequence. Between any two consecutive sequences, approximately 5 s of motor initialization time was inserted. The most important difference from the interfractional change experiment was that a respiratory model initially established by the RPM system was gradually changed during the experiment, resulting in a beam interruption signal produced by the RPM predictive filter. Therefore, the duty cycle was expected to be changed from the initial value (30%). The threshold of the predictive filter was set to be a default value.^(20)^

#### G.4 Evaluation for real patient respiratory motion

To evaluate the capabilities of our system in more realistic situation, additional experiments were performed using the motion platform B in conjunction with two patients' respiratory data. The input data for the platform were made by processing RPM log files that were acquired during the 4D CT simulation and one treatment session of each patient. It should be noted that only anterior‐posterior movement was simulated in this experiment because the RPM system could support vertical tracking only. The RANDO phantom mounted on the platform underwent 4D CT scans while the platform reproduced the patient's simulation session. Finally, the RMVS was tested while the phantom reproduced the patient respiratory motion in treatment room by the same procedures as described above.

## III. RESULTS

### A. Compatibility between RMVS and RPM

The phantom experiment demonstrated that all of the functions of the developed system (i.e., RMVS) were successfully executed. Without any interference the system worked together with RPM as intended. The phase values generated by RPM were successfully transferred to the system with a minimum time delay of approximately 60 ms. A possible source of time delay could be the TCP/IP communication between RPM and RMVS. As the time delay existed consistently, a constant phase shift value was applied to MEC.

### B. Evaluation results for system accuracy and precision

**Figure 6:**
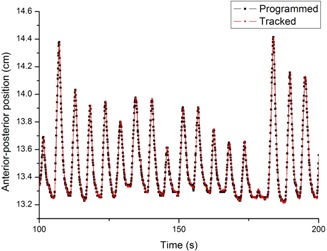
The programmed motion of platform B coincided perfectly with the marker motion obtained from the stereocamera system, confirming the accuracy of the systems.

As seen in Fig. 6, the respiratory curve obtained from the stereocamera system perfectly coincided with that of the input data for platform B. The mean absolute error for 300 s of simulation calculated for a single marker was 0.2 ± 0.2 mm, which validated our previous findings on the accuracy of the system. Table 2 summarized the results of accuracy evaluation for platform A and 4D CT. It was demonstrated that platform A also moved accurately as programmed when compared to the tracking result of the stereocamera system. However, the 4D CT showed a slight underestimate of the displacement, which would be propagated to the error in our system. Details on inaccuracy of 4D CT imaging follows in the Discussion Section below.

**Figure 7:**
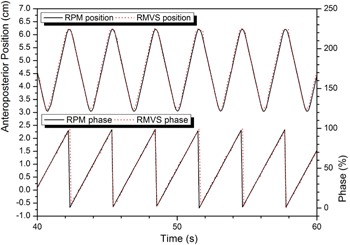
Phase comparison between the RPM and the RMVS. Good agreement in two phase values demonstrated that the phase synchronization was successfully carried out and the selected value for the constant phase offset was optimal.

In phase synchronization, phase values of RMVS coincided well with those of the RPM system, which implied that the PSP worked well with both systems (Fig. 7). The mean absolute error for 180 s calculated for all markers was 1.4 ± 3.5%. It should be noted that a constant phase offset had been applied to the RMVS to account for the systematic time delay.

**Table 2:** Motion parameters of the reference motion independently measured by stereocamera system, 4D CT, and RPM

	*Displacement (mm)*	*Mid position (mm)*	*Cycle (s)*
Programmed	31.0	22.5	3.1
Stereocamera^a^	30.9±0.1	22.3±0.1	3.1±0.1
4D CT^b^	30.3±0.4	22.6±0.1	N/A
RPM^a^ ^,^ c	31.7±0.0	23.2±0.0	3.1±0.0

a Mean value of ten cycles and four marker positions.

b Mean value of four positions corresponding to each marker position on 4D CT.

c The baseline was corrected by using a reference position.

RPM = real‐time position management system; 4D CT = FOUR‐dimensional computed tomography.

### C. Phantom evaluation results

**Figure 8:**
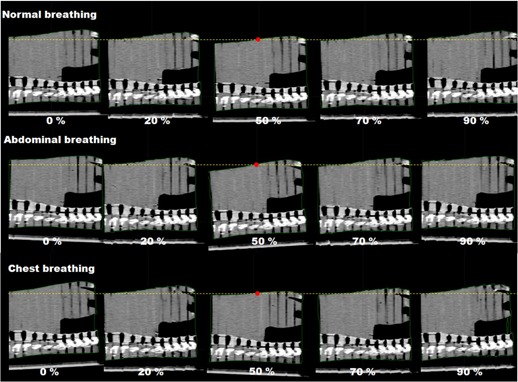
Phantom sagittal images from 4D CT sets acquired during reference motion, abdominal breathing, and chest breathing. The dotted line was set parallel to the middle surface point on the end‐of‐exhale phase image.

Figure 8 shows the 4D CT images of the phantom with the normal breathing. For the comparison purpose, 4D CT images from abdominal and chest breathing motions are also illustrated. It was observed that even though the breathing type changed, the movement of the central region of the phantom was similar to that of the reference breathing, which would be a potential pitfall of single marker‐based monitoring.

#### C.1 Evaluation of interfractional changes in breathing motion

**Figure 9:**
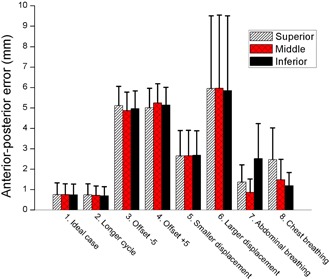
MAEs between positions of the tracked makers and the 4D reference lines in the phantom experiments. The error bars represent 1 standard deviation.

From these experiments, it was confirmed that RPM phase‐based gating didn't provide any warning or interruption signals, even if there were significant interfractional changes in respiratory motion pattern. This supports the usefulness of our system as a respiratory QA tool. For each experiment, MSEs and MAEs between external marker positions and 4D reference lines were calculated. These are summarized in Tables 3 and 4 and graphically shown in Fig. 9. MSE mode was useful to detect baseline shifts with their direction (overall errors of ‐5.0 ± 0.9 and 5.1 ± 0.9 mm for experiments 3 and 4, respectively). In MAE mode, the system detected errors even in the ideal and cycle change cases (overall errors of 0.8 ± 0.5, and 0.7 ± 0.5 mm for experiments 1 and 2, respectively), which turned out to be the systematic error in our system. Relatively large errors and deviations were observed in the displacement change experiments compared to the ideal case (overall errors of 2.7 ± 1.2, and 5.9 ± 3.6 for experiments 5, and 6, respectively). For breathing type change cases, the errors of the middle marker were relatively small (0.9 ± 0.7 and 1.5 ± 1.0 for experiment 7 and 8, respectively), compared to those of the inferior and superior markers (inferior marker error of 2.5 ± 1.7 for experiment 7 and superior marker error of 2.5 ± 1.5 for experiment 8). This result indicated that our multiple marker tracking can detect the abnormal changes in breathing type even when a single marker‐based system misses them.

**Table 3:** MSEs between positions of the tracked makers and the 4D reference lines from the experiment of interfractional motion change

		MSE ± SD (mm)			
*Experiment No*.	*Description*	*Superior*	*Middle*	*Inferior*	*Overall*
1	Ideal case	0.1±0.9	0.0±0.9	0.3±0.9	0.1±0.9
2	Longer cycle	‐0.2±0.9	0.1±0.9	0.0±0.8	0.0±0.9
3	Offset ‐5	‐5.1±0.9	‐4.9±0.9	‐5.0±0.9	‐5.0±0.9
4	Offset +5	5.0±1.0	5.2±0.9	5.1±0.9	5.1±0.9
5	Smaller displacement	‐0.1±2.9	0.0±2.9	0.1±2.9	0.0±2.9
6	Larger displacement	‐0.1±6.9	0.2±7.0	0.1±6.9	0.1±6.9
7	Abdominal breathing	‐0.8±1.4	‐0.6±0.9	‐0.9±2.9	‐0.8±1.9
8	Chest breathing	‐0.2±2.9	0.4±1.8	0.0±1.4	0.1±2.1

MSE = mean signed error; SD = standard deviation.

**Table 4:** MAEs between positions of the tracked markers and the 4D reference lines from the experiment of inter‐fractional motion change

		MAE ± SD (mm)			
*Experiment No*.	*Description*	*Superior*	*Middle*	*Inferior*	*Overall*
1	Ideal case	0.8±0.6	0.8±0.5	0.7±0.5	0.8±0.5
2	Longer cycle	0.7±0.5	0.7±0.5	0.7±0.4	0.7±0.5
3	Offset ‐5	5.1±0.9	4.9±0.9	5.0±0.9	5.0±0.9
4	Offset +5	5.0±1.0	5.2±0.9	5.1±0.9	5.1±0.9
5	Smaller displacement	2.6±1.2	2.7±1.3	2.7±1.2	2.7±1.2
6	Larger displacement	6.0±3.6	6.0±3.6	5.8±3.7	5.9±3.6
7	Abdominal breathing	1.4±0.8	0.9±0.7	2.5±1.7	1.6±1.4
8	Chest breathing	2.5±1.5	1.5±1.0	1.2±0.6	1.2±0.6

MAE = mean absolute error; SD = standard deviation.

**Figure 10:**
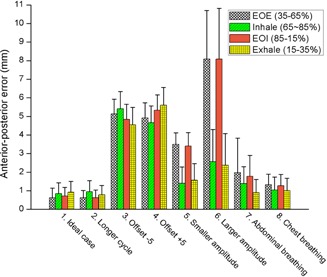
Motion errors analyzed separately for each respiratory phase (end‐of‐exhale (EOE), inhale, end‐of‐inhale (EOI), and exhale). The error bars represent 1 standard deviation.

Figure 10 shows the result of overall motion errors separately calculated for four different phases (i.e., end‐of‐exhale (EOE), inhale, end‐of‐inhale (EOI), and exhale]. This result demonstrated that, for breathing pattern changes related to displacement (experiments 5–8), EOE and EOI are more sensitive to detect such changes than other phases.

#### C.2 Evaluation of intrafractional changes in breathing motion

**Figure 11:**
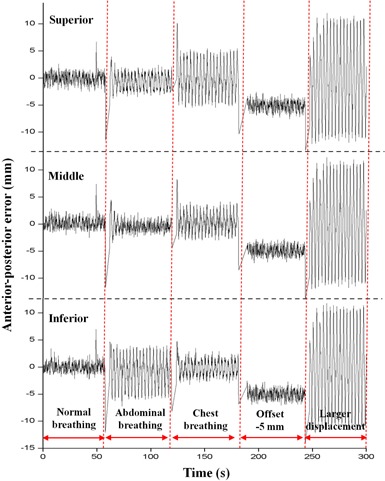
A motion log with signed errors acquired from the intrafractional change experiment. The breathing motion changes could be detected in real time by monitoring positional errors from multiple marker data.

In contrast to the interfractional change experiments, the RPM predictive filter in part provided beam‐off signals in the gating window for the intrafractional changes in breathing motion. However, after a few cycles, the beam‐on signal was resumed. Table 5 summarized duty cycles and motion errors measured during the beam‐on time. The results indicated that our system can report on the significant intrafractional motion changes even when the RPM predictive filter cannot fully handle this problem. Figure 11 shows error logs for each marker as a function of the simulation time.

**Table 5:** Duty cycle and positional error during beam‐on time from the experiment of intrafractional motion changes

			MAE ± SD (mm)			
*Motion Sequence*	*Simulation Time (s)*	*Duty Cycle (%)*	*Superior*	*Middle*	*Inferior*	*Overall*
Normal breathing (4D CT reference motion)	53.9	30.3	0.6±0.5	0.7±0.4	0.7±0.4	0.7±0.4
Abdominal breathing	59.5	26.3	0.9±0.6	0.9±0.6	4.4±1.3	2.0±1.9
Chest breathing	57.9	29.7	3.2±1.6	1.5±0.9	1.2±0.7	2.0±2.0
Offset ‐5	57.0	26.0	5.1±1.3	5.0±1.1	5.1±1.0	5.1±1.1
Larger displacement	57.0	28.4	7.9±2.7	7.9±2.7	7.9±2.7	7.9±2.7

MAE = mean absolute error; SD = standard deviation

#### C.3 Evaluation results for real patient respiratory motion

**Figure 12:**
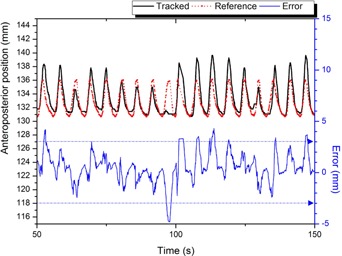
The tracking result of RMVS for a single marker in the experiment with real patient data (patient 1). Irregularities were observed in tracked marker positions (solid black line). Error events (solid blue line) could be detected in real time by using the reference positions from the 4D CT (dotted red line). A tolerance level was set to ± 3 mm for the example purpose (dotted blue line).

**Figure 13:**
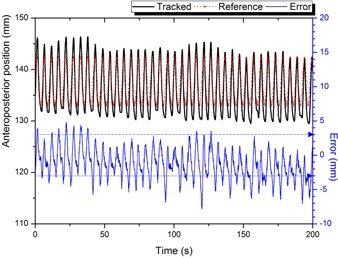
The tracking result of RMVS for a single marker in the experiment with real patient data (patient 2). A significant baseline shift over time was observed in the tracked marker positions (solid black line). As seen in the error curve (solid blue line), this abnormality could be detected by comparing the marker positions with the reference positions from the 4D CT (dotted red line). A tolerance level was set to ± 3 mm for the example purpose (dotted blue line).

While platform B simulated the real patient motion for approximately 300 s, the anterior‐posterior positional errors between the tracked markers and the 4D reference lines were successfully reported by RMVS in real time. Figure 12 demonstrated that patient 1's breathing was rather irregular, but in a stable baseline. On the other hand, a gradual baseline drift was observed in the simulation of patient 2's respiratory data, as seen in Fig. 13. Table 6 summarized the evaluation results.

**Table 6:** MAEs between positions of the tracked markers and the 4D reference lines from the experiments with real patient data

	MAE ± SD (mm) ^a^				
*Patient No*.	*EOE (35%–65%)*	*Inhalation (65%–85%)*	*EOI (85%–5%)*	*Exhalation (15%–35%)*	*Overall*
Patient 1	0.8±0.4	1.1±0.8	1.7±1.2	1.1±0.9	1.2±0.9
Patient 2	1.9±1.4	2.9±1.9	2.2±1.6	1.5±1.1	2.1±1.6

a Mean value of all three markers.

MAE = mean absolute error; SD = standard deviation; EOE = end of exhalation; EOI = end of inhalation.

## IV. DISCUSSION

The assumption of this study was that there would be good correlation between the tumor and the marker movements and that this correlation would be consistent over the entire course of the treatment. Several studies demonstrated good correlation between external marker motion and internal organ movement in free‐breathing respiratory gating treatment.^(^ ^3^ ^,^ ^24^ ^)^ In contrast, some studies reported significant interfractional variability in the internal‐external correlation.^(^ ^2^ ^,^ ^25^ ^)^ Similarly, Hoisak et al.^(26)^ found that intrafractional variations in the tumor‐surrogate relationship occurred less frequently and with smaller magnitude than interfractional variations, which supported the necessity of once‐daily imaging to improve interfractional reproducibility. Therefore, if pretreatment 4D CBCT is available, the developed system together with daily‐basis 4D reference positions can be optimally used to evaluate intrafractional error. In such cases the 4D CBCT can be used to correct the interfractional error and to update the baseline of an internal‐external correlation function.

Venkat et al.^(12)^ proposed a novel method to improve respiratory reproducibility between simulation and treatment. They created a guiding waveform from the RPM signal during CT simulation, and this waveform was used as a visual biofeedback signal during treatment. However, in their study, quantitative evaluation based on treatment room coordinates was not performed due to the limitation of the RPM calculating the 3D position. In addition, placement error of a single RPM marker block can be a source of uncertainty.^(27)^ Major improvements of our technique from the Venkat study include using treatment room coordinates, employing multiple external markers, and introducing the 4D CT‐based reference lines.

In this study, RPM real‐time calculated phase data were transferred to our system. To acquire patient's real‐time phase information, RPM needs an interface to transmit real‐time respiratory signals to third‐party software. However, such a function is not included in the current commercial version of RPM. A special cooperation of the manufacturer is a prerequisite to extract real‐time phase signal from the RPM system.^(12)^ Santoro et al.^(8)^ reported that the RPM real‐time calculated phase data was error‐prone and the retrospective phase calculation using a RPM log file (so‐called “vxp file”) was more accurate. However, this log file can be exported only after the treatment session, and thus the retrospectively calculated phase data cannot be used for the online quality assurance purpose.

It was noteworthy that in the MAE analysis, the experiment 1 (ideal case) exhibited an inherent systematic error of approximately 1 mm. Although there were several potential sources of this error, such as uncertainties of stereocamera‐based tracking, instability of the motion phantom, and so on, it most likely stemmed from the geometric inaccuracy of 4D CT data (as shown in the Results SectionB). Biederer et al.^(28)^ and Hurkmans et al.^(29)^ previously reported that 4D CT scans underestimate the displacement of tumor motion due to their limited temporal resolution. Further study is required to improve the accuracy of the 4D CT.

The results of the phantom experiments indicated that our system could not detect any abnormalities due to the respiratory cycle change alone. The respiratory cycle change leads to a change in transpulmonary pressure, which in turn would potentially cause a change in hysteresis patterns.^(4)^ Since the correlation of organ movement patterns and external surrogate markers is highly dependent on hysteresis patterns, it is inferred that the reproducibility of respiratory cycle is crucial in respiratory gated treatment. It has been reported that an audiovisual biofeedback^(9)^ or audio prompts^(30)^ are effective to help patients to reproduce their planned breathing cycle. We believe that these techniques can be used in conjunction with our system to overcome the limitation related to the respiratory cycle change. The other limitation of our study is the linear interpolation of 4D reference lines at intermediate phases. Future work will address the use of more realistic interpolation methods, such as the B‐spline deformable registration model proposed by Schreibmann et al.^(31)^

In this study, multiple external markers were placed along the body midline, which may raise a question whether the motion of the body midline is representative for that of the entire surface of the abdomen and chest. Recently, Fayad et al.^(32)^ investigated external‐internal correlation for different ROI positions on the body surface. In their study, it was demonstrated that a central region of the anterior body surface exhibits higher correlation than a lateral region, which justified our approach. Further study is in progress using arbitrarily positioned multiple markers to monitor the entire body surface.

## V. CONCLUSIONS

RPM phase‐based gating is vulnerable to interfractional and intrafractional variations in breathing motions, which can lead to the geographic miss in radiotherapy. Utilizing 4D CT simulation data and optical tracking of multiple external markers, we have developed a real‐time quality assurance tool that can verify the positional reproducibility of patients' breathing between the simulation and treatments. The phantom experiments demonstrated that our system was fully compatible with RPM and could quantitatively detect real‐time positional errors from baseline shifts, displacement changes, breathing motion changes, and irregular breathing. The multiple marker‐based tracking also showed a competence to detect errors from breathing motion changes that the single marker‐based tracking might miss.

## ACKNOWLEDGMENTS

This work was supported in part by the SNU Brain Fusion Program Research Grant No. 400–20100049 (2010–2011) and the National Research Foundation of Korea (NRF) grant (800–20120109 and 490–20120026) funded by the Korea government (MEST).
